# Dry January: The best option for public health?

**DOI:** 10.1016/j.eclinm.2022.101279

**Published:** 2022-01-24

**Authors:** eClinical Medicine

As Dry January draws to a close, the growing support for the month-long campaign of abstinence from alcohol is clear. A month of national sobriety appears as far back in the history books as 1942, when Finland instigated “Sober January” (*Raitis tammikuu*) to aid the war effort against the Soviet Union. Organised campaigns of alcohol-free months are now commonplace throughout Europe and the USA, with millions taking part each year. Against this backdrop, global alcohol use is increasing at a worrying rate. A worldwide modelling study published in 2019 in *The Lancet* suggested that alcohol consumption increased by as much as 70% between 1990 and 2017, driven by trends in southeast Asia. Although there are vast geographical differences in alcohol consumption—North Africa and the Middle East have an alcohol intake of about 5–10%—the issue is going global. Dry January and other similar campaigns position themselves in a light-hearted way, targeting the social drinkers of society. Although these individuals are a key public health target, those who are alcohol-dependent and have severe alcohol use disorders are unlikely to benefit from the cheerful positioning of these fun tests of willpower. Additionally, such campaigns feed into the culture of pushing abstinence as the only option to improve health and imply that reaching abstinence can be done by willpower alone. Although the situation for casual drinkers and those with high-risk or severe alcohol use disorders are distinct, our attitudes to alcohol reduction are intertwined for both groups, to the detriment of those at the more severe end of the spectrum.

The harms of alcohol consumption are well established, with alcohol use disorder among the leading causes of morbidity and mortality worldwide. Current WHO documentation states that alcohol consumption is a causal factor in more than 200 disease and injury conditions. In 2021, *The Lancet Oncology* published a study estimating that 4.1% of new cancer cases in 2020 were attributable to alcohol consumption and The Global Burden of Disease Study has shown that the “level of consumption that minimises health loss is zero”.

Treatment for alcohol use disorder can be largely categorised into four main elements—cessation, psychological therapy, moderation, and medicinal treatments. Research suggests that one of the main reasons that people do not seek treatment for alcohol use disorder is that they are not ready to stop drinking completely and do not continue with treatment after an initial referral for this same reason. Although some individuals might be unable to practice moderation, for many there are considerable benefits in reducing consumption. While current clinical guidelines for the treatment of alcohol use disorder and high-risk drinking include both moderation (variably defined by units or blood alcohol concentration) and cessation, the idea that those who are alcohol dependent must become teetotal remains pervasive in the general population and medical community. However, a 2017 article in *The Lancet Psychiatry* showed that a reduction in WHO drinking risk level was associated with significantly lower odds of alcohol dependency 3 years later, especially for very high-risk drinkers. In another study of patients with alcohol use disorder, reductions of one or two WHO drinking risk levels were associated with significant improvements in mental health and quality of life, significant reductions in systolic blood pressure, and improvements in liver enzyme concentrations. A further study in people with alcohol use disorder showed that those who reduced their risk levels by 2.8 units had similar grey matter volumes to complete abstainers, while those who reduced their levels by only 1.2 units had significantly smaller frontal grey matter and thalamic volumes than abstainers and those who maintained reductions of 2.8 units.

One of the key benefits of Dry January and similar campaigns is the normalisation of reducing alcohol intake. In line with the goals of Alcohol Change UK, who instigated Dry January in 2013, for a future in which “people drink as a conscious choice, not a default”, the campaign has increased awareness and raises money to support those with severe alcohol dependencies. However, a 2021 study found that increased Dry January participation in the UK between 2015 and 2018 did not result in a decrease in overall consumption nationwide. Unexpected negatives of abstinence months for casual drinkers include feeling at greater liberty to drink to excess at other times of the year, with binge drinking having a greater damaging effect. Although the public health benefits of the movement towards reducing alcohol intake are important, we must acknowledge the grey areas. We need much more robust evidence to establish the most effective ways to reduce alcohol consumption while providing more adequate support for people with dependencies. Recommendations of stepped or staggered reductions (eg, a few alcohol-free days per week, reducing a unit per week, swapping every other drink for an alcohol-free alternative) could be a more long-lasting solution and appropriate for more people. While the final goal of stepped reductions might be abstinence for some, in this public health crisis of alcohol use, our efforts should also be targeted at supporting and listening to those most affected to help aid in their road to recovery. The shame and disappointment often felt by people with alcohol use disorders who are unable to maintain complete abstinence is greatly unjust. How many people with alcohol use disorders might have lived longer had they been supported with stepped reductions? For the future, we need to focus on making the first steps more accessible to reduce the overall burden of alcohol for good.

